# Improving community ambulation after stroke: the AMBULATE trial

**DOI:** 10.1186/1471-2377-9-8

**Published:** 2009-02-11

**Authors:** Louise Ada, Catherine M Dean, Richard Lindley, Gemma Lloyd

**Affiliations:** 1 Discipline of Physiotherapy, Faculty of Health Sciences, The University of Sydney, Sydney, NSW, Australia; 2 Westmead Hospital, Faculty of Medicine, The University of Sydney, NSW 2006, Australia

## Abstract

**Background:**

It has been reported that following rehabilitation, only 7% of stroke survivors are able to walk at a level commensurate with community participation. Previous research indicates that treadmill and overground walking training can improve walking capacity in people living in the community after stroke. The main objectives of the AMBULATE trial are to determine (i) whether a 4-month treadmill walking program is more effective than a 2-month program, compared to control, in improving walking capacity, health and community participation and (ii) the "threshold" walking speed that results in sufficient walking capacity that makes walking self-sustaining.

**Methods/Design:**

A prospective randomised controlled trial of unsupported treadmill training with a 12 month follow-up with concealed allocation and blinded assessment will be conducted. 210 community-dwelling people after stroke who are able to walk independently but slowly will be recruited and randomly allocated to either a 4 month training group, 2 month training group or the control (no intervention) group. Intervention for the two training groups will occur 3 days per week for 30 minutes each session. Measurements of walking, health and community participation will be taken at baseline, 2 months, 4 months, 6 months and 12 months. This study has obtained ethical approval from the relevant Human Research Ethics Committees.

**Discussion:**

By improving stroke survivors' walking ability, it is likely also to improve their general wellbeing by promoting better health and greater community participation. Furthermore, if stroke survivors can reach a point where their walking and community participation is self-sustaining, this will reduce the burden of care on family and friends as well as the economic burden on the health system. Given the major demographic shift in developed nations involving significant growth in the aged population, this research will make an important evidence-based contribution to the promotion of healthy ageing.

**Trial registration:**

This trial is registered with the Australian New Zealand Clinical Trials Registry, (ACTRN012607000227493)

## Background

Walking is an important human activity which enables us to be productive and participative members of a community. Normal walking speed for older persons is 1.3 m/s [1] and walking capacity measured by the distance covered in 6 minutes is 576 m for men and 494 m for women [2]. After stroke, although the majority of patients leave rehabilitation with some level of independent walking, many have residual walking disabilities. Walking speed of community-dwelling people after stroke has been reported to be around 0.5 m/s with studies reporting a range between 0.3 and 0.8 m/s [3-7]. Walking capacity has also been found to be markedly reduced with 6-minute distance being reported as around 250 m ranging between 40 and 400 m [8,9]. This reduction in walking speed and capacity results in major limitations in community participation. For example, Hill and colleagues [3] found that many individuals after stroke could not walk fast enough to cross the road safely or far enough to do the shopping. The consequence of poor walking ability is widespread, affecting the person and their family and friends. Poor walking ability has been found to reduce quality of life with a reduction in participation in activities outside the home and therefore social isolation. In particular, patients fear dependency more than anything else after a stroke and the ability to walk independently has been found to provide the greatest protection against dependency [10]. A community ambulator is less dependent on family and friends not only for assistance with household tasks but also for assistance with community participation.

**Figure 1:**
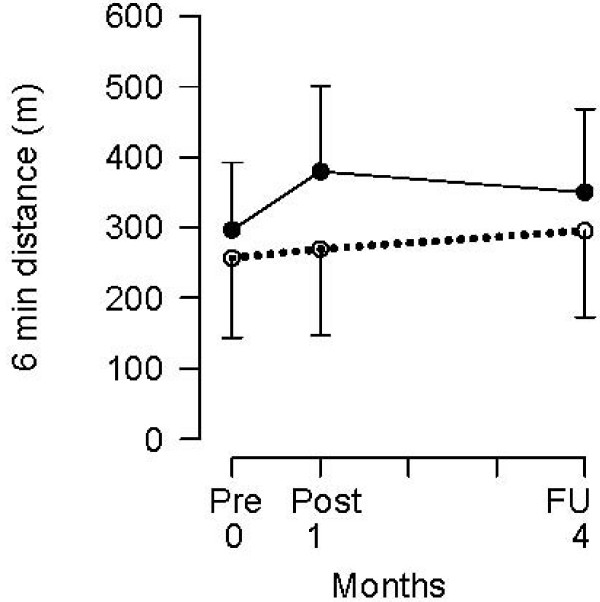
**Improvements of 80 m in 6-min distance following a one-month treadmill and overground walking program (solid = experimental, dotted = control) with subsequent relapse over three months (from Ada et al **[17] with permission).

One of the most promising ways of increasing walking ability post-discharge from rehabilitation after stroke is to use treadmill training. Although the Cochrane review on treadmill training [11] suggests that *supported *treadmill walking is somewhat effective in improving walking (increase in walking speed 0.24 m/s, 95% CI -0.19 to 0.66) in inpatients that can already walk, the review did not examine *unsupported *treadmill training when used post-discharge as a means of improving community ambulation. There have been several uncontrolled trials which suggest that *unsupported *treadmill training is effective. For example, Macko et al [12,13] demonstrated an increase in fitness after a 6-month treadmill training program in community-dwelling ambulatory people after stroke. Smith et al [14,15] demonstrated an increase in strength and a decrease in spasticity after a 3-month program of treadmill training while Silver et al [16] demonstrated an increase in walking speed. In a previous randomised controlled trial of *unsupported *treadmill training in community-dwelling people after stroke, it was demonstrated that a 4 week program is effective in improving walking speed and distance covered in six minutes [17]. However, the gains in 6-minute distance were not maintained at follow-up 3 months later (Figure 1).

In summary, the main objectives of the AMBULATE trial are to determine (i) whether a 4-month treadmill and overground walking program is more effective than a 2-month program, compared to control, in improving walking capacity, health and community participation and (ii) the "threshold" walking speed that results in sufficient walking capacity that makes walking self-sustaining.

## Methods/Design

**Figure 2:**
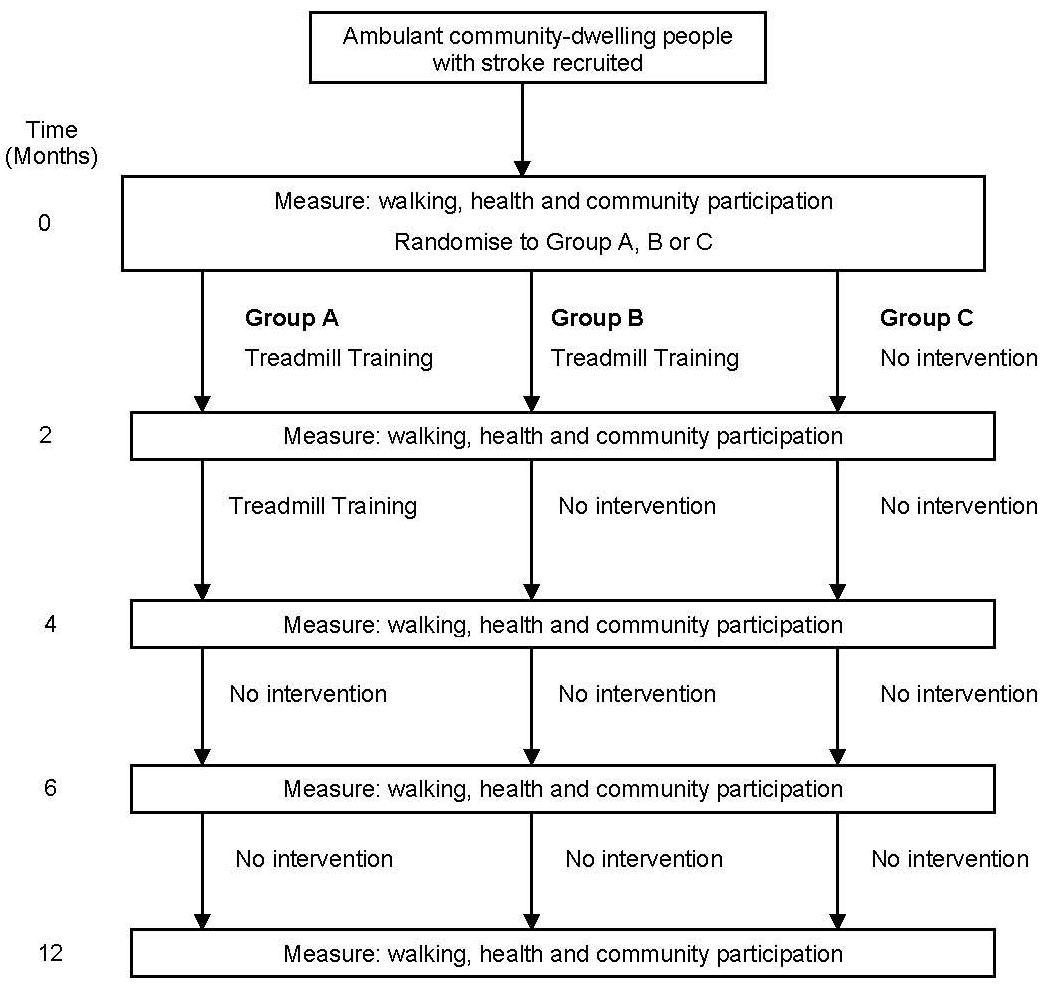
**Design of study**.

A prospective, randomised controlled trial will be carried out (Figure 2). It will include concealed randomisation, blinded assessment and intention-to-treat analysis. 210 participants will be recruited and randomly allocated into either Group A (walking training 3 times per week for 4 months), Group B (walking training 3 times per week for 2 months) or Group C (no active intervention). Outcome measures will be collected at baseline, 2 months, 4 months, 6 months and 12 months. Both data collection and data analysis will be completed by a researcher who is blinded to group allocation. The study has obtained ethical approval from the appropriate Human Research Ethics Committees.

The end point of the training phase of the study for Group A will be 4 months and for Group B will be 2 months. The end point of the follow-up phases of the study will be 6 months and 12 months after admission to the study for all groups.

### Participants

People with stroke will be screened and invited to participate if they:

1. are within 5 years of their first stroke, are adults capable of providing consent, defined as having a Mini Mental State Exam score of > 23 have been discharged from formal rehabilitation and are community-dwelling;

2. walk slowly, defined as 'being able to walk 10 m across flat ground in bare feet without any aids with a speed of less than 1 m/s.

Participants suitable for inclusion in the study need permission granted by their medical practitioner before admission to the study. They will be excluded if they:

1. have an unstable cardiac status which would preclude participation in a treadmill training program (i.e., permission not granted by their medical practitioner), or

2. have severe cognitive and/or language (aphasia) deficits which would preclude them from participation in the training sessions (i.e., unable to follow 2-step commands).

Interpreters will be used for those participants for whom English is difficult.

### Randomisation

Randomisation will be computer-generated, independent and concealed. Participants will be recruited in cohorts of 15. Each cohort will be ranked in descending order according to comfortable walking speed over 10 m and then organized into matched triplets. The participants in each triplet will then be assigned to Group A or B or C by random allocation generated by computer in order to provide secure randomisation, concealed from the recruiter. In this way, participants will be stratified according to initial level of walking disability since this has been found to affect outcome [18].

### Intervention

Training for both intervention groups will be based on a previously-successful treadmill walking program [17]. It will be carried out 3 times a week and the sessions will comprise 30 min of walking. Participants will receive individual training from a physiotherapist; however, there will be opportunity for social interaction since several participants will be trained concurrently. The program will be carried out in a community setting and transport will be provided if necessary. Guidelines will outline the progression of training. Information describing the specific features of the training session (such as treadmill speed, distance walked, assistance required, etc) will be recorded to monitor adherence to the guidelines and to be able to describe the intervention accurately.

Treadmill walking will be structured to increase stride length, speed, workload, and automaticity. To increase stride length, the treadmill will be run at a comfortable speed and participants will be instructed to "walk as slowly as possible". In addition, marching type steps will be included to encourage hip and knee flexion during swing phase to improve toe clearance. A metronome may also be used to decrease cadence and to encourage larger steps. When a normal step length is observed, the speed of the treadmill will be increased (until step length is compromised). Workload will then be addressed by increasing the incline of the treadmill. Finally, automaticity will promoted by presenting the participants with a concurrent cognitive task while maintaining speed and normal step length. The cognitive task may include the participant holding a conversation with the physiotherapist or may be a more structured task such as matching the word "red" with the response "yes" or the word "blue" with the response "no". During treadmill walking, attention will also be focused on alignment to help participants keep their trunk vertical.

The gains achieved during treadmill walking will be reinforced with some overground walking each session, starting with 20% overground walking and working up to a maximum of 50%. Overground walking is defined as whole task practice involving propulsion forwards, backwards, sideways or up and down stairs. To reinforce the increased stride length achieved during treadmill walking, visual cues will be supplied in the form of non-slip footprints which will be laid at intervals normal for that participant's height. As step length approximates normal, participants will be encouraged to walk faster and will be timed for feedback. Participants will also vary the direction of walking to include sideways and backwards, while maintaining vertical trunk alignment. Workload will be increased by introducing stairs and slopes and automaticity will be promoted by the introduction of conversation while walking around an outdoor circuit of curbs, slopes, stairs and rough terrain.

### Measurement

Since the main aim the study is to improve community ambulation, the primary outcome will be walking capacity measured as the distance covered during the 6-minunte walk test at 12 months. Given the hypothesis that improving walking capacity will directly lead to improvements in health and community participation, the secondary outcomes measures will include measures of health and community participation as well as a measure of walking quality.

Therefore, the outcome measures will be:

• Walking

Walking capacity will be assessed using the 6-minute Walk Test (distance covered in 6 min). Walking quality will be assessed using the 10-metre Walk Test (speed, stride length, cadence).

• Health

Health will be assessed by recording number of falls, number of readmissions to hospital, number of comorbidities, number of visits to the doctor, and quality of life (Euroqol EQ-5D score).

• Community participation

Community participation will be assessed using a self-efficacy questionnaire about walking ability, based on the Falls Efficacy Scale [19] and the Adelaide Activities Profile [20].

### Sample size

210 participants will be recruited. The sample size has been calculated to reliably detect a treatment effect size of a 50 m difference in walking capacity with 80% power at a two-tailed significance level of 0.05 on any pairwise comparison. This effect size has been derived from the walking capacity of a population of community-dwelling stroke patients who took part in the previous treadmill walking program [17]. On entry to this randomised controlled trial, the distance walked in 6 minutes was 280 (SD, 100) m using the same measurement procedure to the present proposal. The smallest number of participants to detect a 50 m difference between two groups estimated from independent samples is 63 participants per group, i.e., 189 participants in total. On the assumption that about 10% of the participants may drop out during the course of the study, we have set a target of 210 participants in total.

### Statistical analysis

The variables that reflect walking capacity, community participation and quality of life will be analysed by pairwise comparisons between the three groups using the Students' t-test, or Wilcoxon's rank-sum test if it is clearly not normally distributed.

The other health variables: number of falls, number of readmissions to hospital, comorbidities, and number of visits to the doctors will be expressed as a proportion of events per person and analysed by pairwise comparisons between the three groups using the Chi square test.

To ascertain the threshold of post-intervention walking speed that results in the maintenance of 80% of predicted 6-min distance at 12 months, receiver operating characteristic (ROC) curves will be constructed. Youden's Index will be used to determine the cut-off point for walking speed at the end of the programs that gives the best combination of specificity and sensitivity.

## Discussion

This research has the potential to make a large difference to the quality of life of many stroke survivors. By improving stroke survivors' walking ability, it is likely also to improve their general wellbeing by promoting better health and greater community participation. Furthermore, if stroke survivors can reach a point where their walking and community participation is self-sustaining, this will reduce the burden of care on family and friends as well as the economic burden on the health system. Given the major demographic shift in developed nations involving significant growth in the aged population, this research will make an important evidence-based contribution to the promotion of healthy ageing.

## Competing interests

The authors declare that they have no competing interests.

## Authors' contributions

LA and CD conceived this study. LA, CD and RL contributed to the design of the study and the procurement of funding. CD, LA and GL developed procedures for implementing the protocol. All authors drafted the manuscript and have read the final manuscript.

## Pre-publication history

The pre-publication history for this paper can be accessed here:


